# Cluster of Nipah Virus Infection, Kushtia District, Bangladesh, 2007

**DOI:** 10.1371/journal.pone.0013570

**Published:** 2010-10-21

**Authors:** Nusrat Homaira, Mahmudur Rahman, M. Jahangir Hossain, Nazmun Nahar, Rasheda Khan, Mostafizur Rahman, Goutam Podder, Kamrun Nahar, Dawlat Khan, Emily S. Gurley, Pierre E. Rollin, James A. Comer, Thomas G. Ksiazek, Stephen P. Luby

**Affiliations:** 1 International Centre for Diarrhoeal Disease Research, Bangladesh (ICDDRB), Dhaka, Bangladesh; 2 Institute of Epidemiology Disease Control and Research, (IEDCR), Dhaka, Bangladesh; 3 Special Pathogens Branch, Centers for Disease Control and Prevention (CDC), Atlanta, Georgia, United States of America; University of Texas Medical Branch, United States of America

## Abstract

**Objective:**

In March 2007, we investigated a cluster of Nipah encephalitis to identify risk factors for Nipah infection in Bangladesh.

**Methods:**

We defined confirmed Nipah cases by the presence of IgM and IgG antibodies against Nipah virus in serum. Case-patients, who resided in the same village during the outbreak period but died before serum could be collected, were classified as probable cases.

**Results:**

We identified three confirmed and five probable Nipah cases. There was a single index case. Five of the secondary cases came in close physical contact to the index case when she was ill. Case-patients were more likely to have physical contact with the index case (71% cases versus 0% controls, p = <0.001). The index case, on her third day of illness, and all the subsequent cases attended the same religious gathering. For three probable cases including the index case, we could not identify any known risk factors for Nipah infection such as physical contact with Nipah case-patients, consumption of raw date palm juice, or contact with sick animals or fruit bats.

**Conclusion:**

Though person-to-person transmission remains an important mode of transmission for Nipah infection, we could not confirm the source of infection for three of the probable Nipah case-patients. Continued surveillance and outbreak investigations will help better understand the transmission of Nipah virus and develop preventive strategies.

## Introduction

Among the 122 Nipah cases identified between 2001 to 2007 in Bangladesh, 87 (71%) died and 62 (51%) developed illness following person to person transmission [1]. One of the distinct features of Nipah virus epidemiology in Bangladesh is that only certain case-patients apparently spread the disease to others. In a previous review of cases in Bangladesh, we identified only nine Nipah spreaders and each of them spread the disease to a mean of seven persons (range 1–22). All of the Nipah spreaders died [1]. Though human-to-human transmission plays an important role in subsequent transmission of Nipah [2], [3], in Bangladesh some of the identified routes of introduction of Nipah virus from its natural reservoir, *Pteropus f*ruit bats, in to humans are though drinking of raw date palm sap contaminated by bats, contact with infected animals and possibly through direct contact with bat secretion[4].

Nipah virus has been isolated from human saliva, urine, nasal and pharyngeal secretions [5], [6], [7], [8]. Nipah case-patients with difficulty breathing were more likely to spread the virus (12% versus 0% P = 0.03) [1]. Findings from outbreak investigations in Bangladesh demonstrate that family members, friends, relatives and neighbors who came in direct contact with infected respiratory and other body secretions of Nipah spreaders were significantly at greater risk of subsequently acquiring the infection [2], [3].

In April 2007, a joint investigation team formed from the Institute of Epidemiology, Disease Control and Research (IEDCR) and the International Centre for Diarrhoeal Disease Research, Bangladesh (ICDDR, B) investigated a cluster of fatal encephalitis in a village of Sadar Upazila (sub-district) of Kushtia District. The objectives of the investigation were to identify the cause of the outbreak and the risk factors for development of illness.

## Methods

### Case Definition and Identification

We defined suspect case patients as persons having fever with headache and/or cough or persons having fever with new onset of altered mental status or seizures residing in the outbreak area with an onset of illness during March and April, 2007. We identified suspect case-patients by collecting information from the local health workers in the community and by asking community residents if they were aware of anyone meeting the suspect case-definition in the affected community. We also investigated all deaths in that community in that time period. We asked family members of the decedent, if the decedent had symptoms compatible with the suspect case definition prior to death. We used structured questionnaires to record history of illness and general information about exposures for each suspect case-patient. The team requested the local health authority in the outbreak area to report to the IEDCR if any case-patient with fever and altered mental status came to the local health facility for treatment.

The team collected blood samples from living suspect case-patients. The samples were centrifuged in the field then transported on wet ice to the laboratory at IEDCR where they were stored at −70°C. We tested the samples at IEDCR with an immunoglobulin M (IgM) capture enzyme immunoassay that detects Nipah IgM antibodies[9]. The samples were then confirmed at Centers for Disease Control (CDC), Atlanta using IgG and IgM capture enzyme immunoassay. We categorized suspect case-patients who had laboratory evidence of acute infection, shown by presence of IgM and IgG to Nipah virus in serum, as confirmed cases. Suspect case-patients who died and who resided in the same village as confirmed case-patients during the outbreak period were classified as probable Nipah case-patients because no specimen was available as the patient died before the investigation was initiated.

### Qualitative Study

A team of two Bangladeshi anthropologists conducted in-depth interviews with living confirmed and probable case-patients and with family members who cared for cases, including the deceased case-patients. They also conducted informal discussions with neighbors of case-patients with the objective of exploring possible modes of transmission of disease.

### Case-control Study

The field team enrolled confirmed and probable case-patients in a case-control study to investigate exposures associated with Nipah infection, including person-to-person transmission. We selected three neighborhood controls for each case-patient, starting from the fourth closest courtyard to the case-patient to reduce over matching of demographic characteristics of neighboring household, confirming that no members in the households of that courtyard were ill during the outbreak period. Each courtyard had two to three households and we selected only one household from each courtyard which was closest to case-patient's household. The household resident closest in age to the case-patient was eligible to participate as a control. We selected controls closets in age to the cases not to control for confounding effect of age but to provide interviewers with easy guidelines to select controls. The field team selected only one control from each courtyard. This process was repeated at the next closest household until we enrolled three controls for each case.

We used proxy respondents for each case-patient who was too sick to answer, or who had died. Proxy respondents included family members and friends who were aware of the case-patients activities and probable risk exposures in the 30 days preceding their illness. Multiple proxy respondents were used to ensure maximum reliability of the information as friends or colleagues might be more informed about possible exposures outside the home whereas family members were likely to be better informed about domestic exposures. The field investigation team used a standardized structured questionnaire to collect information on demographics, signs and symptoms of illness and possible risk factors for Nipah transmission such as: exposure to ill patients, including touching, staying in the same room, feeding, sharing a bed or cleaning body secretions; exposure to sick animals in the surrounding area; history of climbing trees; sighting bats near area of residence or working place, contact with bats, eating fruits picked from the ground; and drinking raw date palm sap.

### Statistics

We used descriptive statistics to analyze the socio-demographic and clinical profiles of case-patients. For the case-control study, we used an unmatched analysis because neighbors were chosen as controls to ensure that controls and case-patients were representative of the same population, not to control for confounding factors. We enrolled persons closest in age as controls, not to control confounding by age, but rather to provide simple standardized guidelines to the interviewers for control selection. We used odds ratios (OR) to estimate the association of each exposure with disease and calculated 95% confidence limits (CI) around the odds ratio. To assess that observations were not only due to chance, we used the chi square test when expected cells were ≥5 and the Fisher exact test when expected cell sizes were <5. We considered any association to be statistically significant if the p value was <0.05. To test the hypothesis that Nipah virus was transmitted from the index case to other case- patients, we excluded the index case in all the analyses of person-to-person transmission.

### Ethics

All human study participants gave informed verbal consent for participation in this investigation. The Ethical Review Committee at ICDDR, B reviewed and approved a protocol for encephalitis surveillance and outbreak investigation. As the study was part of an emergency outbreak investigation, approval from an Institutional Review Board was not required.

## Results

### Descriptive findings

The team collected 13 serum samples from 19 suspect case-patients. Seven of the serum samples were collected within less than seven days of illness onset. Three of the four serum samples that were collected after 10 days of illness onset had detectable IgM and IgG Nipah virus antibodies and were identified as confirmed cases. Five (26%) of the 19 suspected case-patients, who resided in the same outbreak village and died during the outbreak period but before samples could be collected, were categorized as probable cases. We also identified one additional death in the outbreak area but the decedent had chronic abdominal problem and had severe abdominal pain before death. He was not included into the study as the deceased did not have symptoms compatible with the case-definition.

**Figure 1:**
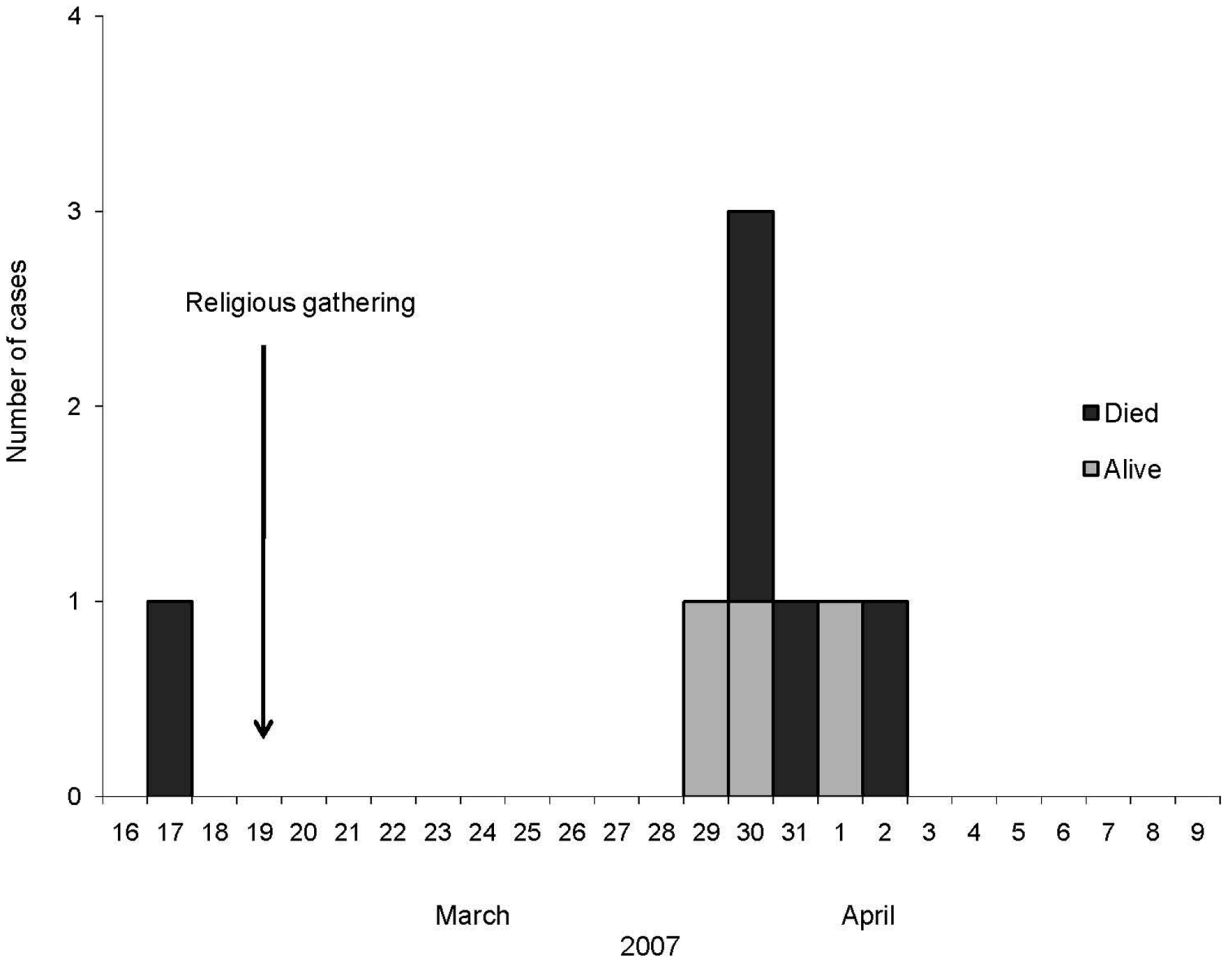
Distribution of Nipah cases by date of onset of illness, March-April 2007.

All the cases were clustered in time. The index case developed illness on March 17, 2007, followed by a single secondary wave of illness 12–16 days later (Figure 1). The mean age of the case-patients was 38 years (range 27–55 years) and 25% were males. The mean duration between illness onset and death was 4 days (range 1–7 days) (Table 1). Among the eight Nipah case-patients; two confirmed and four probable case-patients had fever with altered mental status and or respiratory difficulty.

**Table 1:** Characteristics of Nipah case-patients, Sadar Upazila, Kushtia District, Bangladesh, March-April 2007.

Characteristics	N = 8 (%)
Age	
Mean (SD) in yrs	38 (9)
Median (range)	35 (27–55)
Male	2 (25%)
Clinical features	
Fever	8 (100)
Severe fatigue/weakness	7 (87)
Headache	6 (75)
Vomiting	5 (63)
Cough	5 (63)
Respiratory distress	5 (63)
Muscle pain	5 (63)
Altered mental status	4 (50)
Restlessness	4 (50)
Unconscious *	3 (38)
Joint pain	3 (38)
Case fatality	5 (63)
Onset of illness to death (n = 5), Mean (range)	4 (1–7)

*a subset of patient who developed altered mental status also developed unconsciousness.

### Exposure and illness history

Proxies reported that the index case, Patient A (a 55 year old woman, probable case), developed illness on March 17, 2007 and died on the sixth day of illness on March 22, 2007. On the first two days of illness Patient A developed fever, headache, cough, muscle pain and joint pain which progressed to restlessness, difficulty in breathing, vomiting, altered mental status, spitting of blood tinged saliva and loss of consciousness over the last four days of illness. Patient A reportedly did not have any exposure to identified risk factors for Nipah infection, including drinking raw date palm sap or partially bat eaten fruits, contact with other Nipah patients, climbing trees or contact with bats or sick animals. Five of Patient A's caregivers (Patient B, C, D, E and F) subsequently developed Nipah compatible illness.

Patient B (confirmed case), a friend, took care of Patient A during the entire illness episode; she slept with her at night, carried her to the doctor, cooked food for her, doused her head with water several times, cleaned her vomit, bathed her and shared rice with her from the same plate. Patient B developed Nipah illness 8 days after Patient A died (duration of exposure to illness onset 8–13 days). Patient C (probable case), another friend, also took care of Patient A and slept with her during the last three days of her illness; she developed illness within 11 days of contact with Patient A (duration of exposure to illness onset 9–11 days). Patient C died after seven days of illness. Patient D (confirmed case), a neighbor of Patient A, massaged the index case's head with oil and cleaned her oral secretions during her last two days of illness when she developed breathing difficulty, altered mental status and began spitting. Patient D became ill within 12 days of contact with Patient A. Another neighbor of Patient A, Patient E (confirmed case), reported that she only touched the hand of the index case once, to check for fever, and contracted the illness 12 days after illness onset and seven days after death of the index case. We do not know on which day of illness or how many times she visited Patient A during her illness episode. Patient F (probable case), Patient A's brother-in-law, only came in physical contact with Patient A on the third day of her illness. He became sick 11 days after contact with the index case and died after six days of illness. None of these five subsequent cases (Patients B-F) had exposure to any other Nipah patient or patient with fever and altered mental status other than Patient A. They also did not have any history of drinking raw date palm sap or coming in contact with any bat or other sick animal.

The grave of a Muslim religious leader, who died in 2001, is in the outbreak village and his followers from different villages gather annually for an anniversary ceremony. On the third day of the index case's illness, on March 19, 2007, this religious ceremony was in the courtyard adjacent to the grave of the religious leader. Although the index case was quite ill at this time, with a severe cough and a varying level of consciousness, she still attended the ceremony. All the other seven case-patients (case-patients B to H) also took part in the ceremony. During the ceremony, the index case lost consciousness. A group of people, including Patient B, C, and D along with other unidentified women, carried her back to her home. We could not confirm if any other men apart from Patient D helped in carrying her home.

Proxy respondents of the two probable case-patients, Patients G and H, who were present at the religious gathering, did not recall observing any physical contact between Patient G or H and the index case in the religious gathering. Proxy respondents also did not report any contact with other Nipah cases, contact with anyone with fever and altered mental status or seizures or contact with sick or dead animals prior to their illness onset. They also did not have any history of possible exposure to environmental risk factors for Nipah infection. Patient G and H both developed illness 11 and 14 days after attending the religious ceremony. Patient G first developed fever and headache with generalized weakness. On the third day of illness she developed severe respiratory distress and died on the same day. Patient H reportedly only developed fever, headache, restlessness and severe weakness. After one day of illness he was taken to a hospital where he died before admission. However, the manager of the place where Patient H worked reported that four days before his death he had borrowed some money saying that he was not feeling well and needed to seek medical care, but the manager did not know the details of his illness.

### Case-control study

We enrolled the three confirmed and five probable cases and three unmatched controls for each case-patient in a case-control study. We used proxy respondents for six case-patients (6/8). Case-patients and controls were similar in age (mean age 38 years SD±9 in cases versus 39 years with SD±10 in controls, p = 0.9) and sex (25% male cases versus 21% male control, OR = 1, 95% CI 0.19–8.30, p = 1).

The only risk factor associated with acquiring Nipah infection was having physical contact (including touching, sharing the same bed and cleaning body secretions) with the index case during illness (71% cases versus 0% controls, odds ratio undefined, p = <0.001) in the bivariate analysis. Though we identified two bat roosts within three and six kilometers from the outbreak area, there were no significant associations between illness and drinking raw date palm sap, climbing trees, sighting bat in the area of residence or working place, eating fruits picked from the ground or having contact with bats or sick animals (Table 2).

**Table 2:** Bivariate analysis of risk factors for Nipah infection, Sadar Upazila, Kushtia District, Bangladesh, March-April 2007.

Risk Factor	Cases		Controls		OR (CI)	P value
	Number	%	Number	%		
Male sex	2	25%	5	21%	1 (0.19–8.3)	1
Climbed trees	0	0%	1	4%	undefined	1
Physical contact with living animals						
Cow	5	62.5%	11	46%	1.9(0.4–10)	0.7
Dog	2	25%	3	13%	2.3 (0.3–17)	0.6
Drank raw date palm sap	0	0%	0	0%	undefined	0.25
Climbed any tree	0	0%	1	4.1%	undefined	1.00
Had contact with bats	0	0%	0	0%	Undefined	0.25
Had seen bats in around place of residence at night	1	12.5%	2	8.3%	1.6(0.12–20)	1.00
Physical contact with the Index case	5	71%	0	0%	undefined	0.000

## Discussion

The findings of this outbreak investigation, specifically the epidemic curve with a single secondary peak of illness supported by a strong association between contact with index case and illness provide compelling evidence for person-to-person transmission of Nipah infection from the index case to the subsequent cases. In this outbreak, five of the subsequent case patients were directly involved in caring for the index case when she was sick. The five subsequent cases developed illness within 8–13 days of contact with the index case which is consistent with the incubation period of human Nipah virus infection observed in other settings [10]. Similar transmission of Nipah virus has been documented repeatedly and probably results from contact with infected respiratory and possibly other body secretions of Nipah patients while caring for them [2], [3], [11].

A unique finding and a major limitation of this investigation is that the index cases and patient G and H did not have definite history of identified risk factors. These three case-patients did not have any history of consuming raw date palm sap within one month prior to death which has been implicated as a possible route of transmission of Nipah virus directly from its natural reservoir in Bangladesh [12]. There was also no history of contact with bat secretion or contact with sick animal [13]. Though we could not ascertain close physical contact of patient G and H with index case, it is possible that they had direct contact with contaminated respiratory droplet or a fomite from the index case. The onset of illness spanned over 17 days for these three cases suggesting that their exposure was not simultaneous, and that if this were direct transmission from bats there were multiple transmission events. Patient G had symptoms similar to Nipah illness[8]. Patient H reportedly only had fever and headache and was included into the study due to our broad cases definition. However, only fever and headache are usually considered harmless by Bangladeshi community residents who seek hospital care only when their health condition becomes serious[11]. Though we will never know the exact cause of death of Patient G and H, sudden death of a previously healthy individual is a rare event and clustering of the deaths in time and place during a Nipah outbreak suggests that Nipah infection was a likely cause of the deaths.

Continued surveillance and outbreak investigations will help in better understanding of transmission of Nipah virus from bats to humans and then from humans to humans. Behavior change communication promoting feasible steps, to avoid unprotected contact with respiratory and oral secretions, such as adequate hand washing or the use of respiratory barriers, while caring for patients with respiratory symptoms both at family and hospital levels could minimize spread of the disease by limiting person-to-person transmission.
